# 523 Weight Loss in Patients with Large Burns

**DOI:** 10.1093/jbcr/irae036.158

**Published:** 2024-04-17

**Authors:** Caran Graves, Callie M Thompson, Christopher R LaChapelle, Irma D Fleming, Giavonni M Lewis

**Affiliations:** University of Utah Health Burn Center, Salt Lake City, UT; University of Utah Health, Salt Lake City, UT; University of Utah, Salt Lake City, UT; University Of Utah Burn Critical Care, Salt Lake City, UT; University of Utah Health Burn Center, Salt Lake City, UT; University of Utah Health, Salt Lake City, UT; University of Utah, Salt Lake City, UT; University Of Utah Burn Critical Care, Salt Lake City, UT; University of Utah Health Burn Center, Salt Lake City, UT; University of Utah Health, Salt Lake City, UT; University of Utah, Salt Lake City, UT; University Of Utah Burn Critical Care, Salt Lake City, UT; University of Utah Health Burn Center, Salt Lake City, UT; University of Utah Health, Salt Lake City, UT; University of Utah, Salt Lake City, UT; University Of Utah Burn Critical Care, Salt Lake City, UT; University of Utah Health Burn Center, Salt Lake City, UT; University of Utah Health, Salt Lake City, UT; University of Utah, Salt Lake City, UT; University Of Utah Burn Critical Care, Salt Lake City, UT

## Abstract

**Introduction:**

The deleterious effect of weight loss in those with burns is well known. However, guidelines for monitoring loss are limited. This descriptive study evaluated weight loss incidence in patients with large burns admitted to a regional burn center.

**Methods:**

Medical records for adult patients admitted to a regional burn center (2019-2021) with burns ≥20% total body surface area burned (TBSA) were reviewed. Patients with amputations were excluded. Data collection included burn size/depth, resuscitation fluids (pre- and post-admission), initial and final weight (last week of admission) and nutritional interventions. Patients were divided into three groups based on %TBSA: 20-29%, 30-39% and ≥40%.

**Results:**

From 2019-2021, 722 patients with new burn injuries were admitted of whom 542 were over 18 years of age. Fifty-two (94% male) met inclusion criteria. Those with smaller burns had fewer surgeries and shorter stays. (Table 1).

Most patients (85%) received resuscitation fluids prior to admission (PTA) with an average of 2782 mL PTA and a total of 21,079 mL after admission. Larger burns received high resuscitation volumes both pre- and post- admission.

Over half of patients lost weight though 23 had a net weight gain. Most patients (56%) lost some weight but nearly half (48%) discharged within 5% of admission weight. The average loss across all groups was only 2.6% of admission weight (Table 2). Patients with larger burns were more likely to lose weight and also had more weight loss.

Almost all (94%) patients required enteral nutrition and all transitioned to full oral intake prior to discharge; no one required parenteral nutrition. Again, patients with larger burns required more enteral intake.

**Conclusions:**

Most patients lost weight though some had net weight gain. Those with larger burns lost more weight than those with smaller burns. Weight loss is a risk factor for malnutrition even with nutritional support. Weight loss in burn patients should be monitored—particularly in those with burns >30% TBSA--and be considered as an outcome measure in burn care.

**Applicability of Research to Practice:**

Weight loss in burn patients should be monitored and considered as an outcome measure in burn care.

**Table:** 

Variable	≥20% TBSA (n= 52)	20-29% TBSA (n = 27)	30-39% TBSA (n = 15)	≥ 40% TBSA (n=10)
Age				
Average Median Range	45.7 47 22-79	45.7 47 22-79	46.2 46 19-66	35.3 29.5 18-65
Sex (% male)	94%	74%	80%	70%
average % TBSA Total Partial Full	32.7% 20.4% 10.7%	23.7% 19.7% 3.9%	34.7% 23.2% 11.6%	54.2% 23.0% 29.6%
LOS (days), average range	56.3 16-165	35.4 16-84	45.6 17-81	79 41-165

**Table:** Table 2. Resuscitation fluids, weight changes, and nutritional support by burn size

	≥20% TBSA (n = 52)	20-29% TBSA (n = 27)	30-39% (n = 14^a^)	≥ 40% TBSA (n=10)
Lost weight n (%)	29 (56%)	10 (37%)	10 (71%)	7 (70%)
Average weight change from admission (range)	-2.5% (-20% to +20%)	+3% (-12.8% to+20%)	-8.8% (-13.1% to+7%)	-7.1% (-29% to +5%)
Nutrition (%) Oral Enteral parenteral	100% 94% 0	100% 85% 0	100% 93% 7%	100% 100% 0
Resuscitation fluids None/unknown Pre- Post- Total	7 2686 mL 18223 mL 20417 mL	7 662 mL 13560 mL 15099 mL	0 3089 mL(n=14) 18370 mL 21253 mL	0 2928 mL 30598 mL 33525 mL

a One with no discharge weight

